# Multiplex real-time PCR FilmArray performance in the diagnosis of meningoencephalitis: lights and shadows

**DOI:** 10.1007/s15010-023-02076-x

**Published:** 2023-07-29

**Authors:** Néstor López, Genoveva Cuesta, Sara Rodríguez-Vega, Enric Rosas, Mariana Chumbita, Climent Casals-Pascual, Laura Morata, Andrea Vergara, Marta Bodro, Jordi Bosch, Sabina Herrera, Jose Antonio Martínez, Josep Mensa, Carolina Garcia-Vidal, María Ángeles Marcos, Jordi Vila, Alex Soriano, Pedro Puerta-Alcalde

**Affiliations:** 1 Infectious Diseases Department, Hospital Clínic-IDIBAPS, Barcelona, Spain; 2 Microbiology Department, Hospital Clinic, University of Barcelona, ISGLOBAL, Barcelona, Spain; 3 Hospital Universitario de Cabueñes, Gijón, Spain; 4 CIBERINF, CIBER Infectious Diseases, Madrid, Spain; 5 Universitat de Barcelona (UB), Barcelona, Spain

**Keywords:** Meningoencephalitis, Meningitis, Encephalitis, BioFire, Multiplex real-time PCR

## Abstract

**Purpose:**

We aimed to evaluate the performance of the FilmArray (FA) meningitis/encephalitis (ME) panel. Secondarily, we analyzed the false positive (FP) and false negative (FN) results, as well as the predictive values of the technique, regarding the cerebrospinal fluid (CSF) characteristics.

**Methods:**

FA is a multiplex real-time PCR detecting 14 of the most common ME pathogens in CSF. All FA performed at our hospital (2018–2022) were retrospectively reviewed. FA was compared to conventional techniques and its performance was assessed based on the final diagnosis of the episode.

**Results:**

FA was performed in 313 patients with suspicion of ME. Most patients had altered mental status (65.2%) and fever (61%). Regarding CSF characteristics, 49.8% and 53.7% presented high CSF proteins and pleocytosis, respectively. There were 84 (26.8%) positive FA results, mainly for HSV-1 (10.9%), VZV (5.1%), Enterovirus (2.6%), and *S. pneumoniae* (1.9%). In the 136 cases where both FA and routine methods were performed, there was a 25.7% lack of agreement. We identified 6.6% FN results, but 28.6% FP, mainly due to HSV-1. This resulted in a high negative predictive value (NPV) of 93.4%, but a positive predictive value (PPV) of 73%. Remarkably, PPV as low as 36.9%, and 70.2%, were found in cases without pleocytosis, or lack of high CSF protein levels, respectively.

**Conclusion:**

FA was associated with high NPV, but frequent FP results and low PPV, particularly for HSV-1, and especially in patients without high CSF protein levels or pleocytosis.

## Introduction

Acute meningoencephalitis (ME) is a life-threatening infection of the central nervous system (CNS) with a remaining high global incidence [1] and up to 19.9% risk of sequelae [2]. Early microbiological diagnosis is essential to guide correct antimicrobial treatment [3], and also to avoid the costs and toxicity of unnecessary treatments. However, conventional microbiological techniques to diagnose ME remain slow and frequently low-yielded [4].

In the last decade, multiplex real-time PCRs to detect the most common microorganisms associated with ME in a cerebrospinal fluid (CSF) sample have been developed for a rapid diagnosis. FilmArray® (FA) ME Panel (BioFire Diagnostics) is a multiplex real-time PCR that detects 14 of the most common bacterial, viral, and fungal causes of infectious ME, with a turnaround time of 3 h [5]. BioFire FA reports an overall sensitivity and specificity of 94.2% and 99.8%, respectively [6]. Nevertheless, a recent meta-analysis including 8 studies and 3059 patients, reported estimates of sensitivity of 90% (95% confidence interval [CI] 86–93%), and a specificity of 97% (95% CI 94–99%) [7]. A later meta-analysis reported a sensitivity of 92.1% (95% CI 86.8–95.3%) and a specificity of 99.2% (95% CI 98.3–99.6%) for bacterial meningitis, based on the analysis of 15 studies including 5524 patients, and considering a final diagnosis adjudication based on clinical/laboratory criteria [8]. In the same meta-analysis, sensitivity for Herpes simplex virus 1 (HSV-1) decreased to 78.2% (95% CI 58.1 − 90.3%) based on 3 studies including 6883 patients. Additionally, there was a concern for potential false positive (FP) and false negative (FN) results reported in real-life practice [7, 8].

We aimed to assess the FA performance since its introduction in the routine practice in our hospital to patients in whom ME was suspected. Secondarily, we analyzed the experienced FP and FN results, as well as the predictive values of the technique regarding the CSF characteristics.

## Methods

### Setting and data collection

This is a retrospective cohort study performed at the Hospital Clinic in Barcelona (Spain), an 800-bed university center that provides broad and specialized medical, surgical, and intensive care attention for an urban population of 500,000 people. Since 2018, our center has been performing FA testing when it was clinically indicated under the suspicion of ME.

### Study population and design

We analyzed all consecutive cases of suspected ME in which FA was performed between January 2018 and February 2022. The following data were obtained for all patients: age and sex, pre-existing immunosuppression, clinical presentation, prior antibiotic therapy, CSF characteristics, intensive care unit (ICU) admission, and 30-day mortality. All data gathered were anonymously registered in a specific database designed for this study.

### Definitions

Prior antibiotic therapy was defined as any antibiotics the patient had received in the last 24 h before obtaining a CSF sample. Prior corticosteroid use was defined as a minimum dose of 0.3 mg/kg/day of prednisone equivalent for > 3 weeks. Neutropenia was defined as an absolute neutrophil count of < 500 cells/mm^3^. Hypoglycorrhachia was defined as CSF glucose levels < 40 mg/dL. High protein CSF levels were > 600 mg/dL, high LDH levels were > 40 U/L, and high lactate levels were > 35 mg/dL. CSF pleocytosis was defined as > 10 leukocytes/mm^3^. The final diagnosis of the episode was determined by the investigators (N.L., G.C., and P.P-A.) after a thorough evaluation of microbiological and radiological results, clinical evolution, response to treatment, and the presence or absence of an alternative diagnosis.

### Microbiological methods

For the FA analysis, 200 µL of CSF was used. Samples were handled in a biosafety cabinet according to the manufacturer's instructions. BIOFIRE® FILMARRAY® Meningitis-Encephalitis (ME) Panel (BioFire Diagnostics, Salt Lake City, USA.) (FA) detects the following 14 potential ME pathogens in CSF: *Escherichia coli* K1, *Haemophilus influenzae*, *Listeria monocytogenes*, *Neisseria meningitidis*, *Streptococcus agalactiae*, *Streptococcus pneumoniae*, Enterovirus, HSV-1, Herpes simplex virus 2 (HSV-2), Human herpesvirus 6 (HHV-6), Human parechovirus, Varicella-zoster virus (VZV), Cytomegalovirus (CMV), and *Cryptococcus neoformans/gattii.*

The routine microbiology protocol for CSF samples with suspicion of ME includes inoculation of blood and chocolate agar, and thioglycolate broth, and *Cryptococcus neoformans* antigen (Remel™ Cryptococcus Antigen Test Kits; Thermo. Scientific, Lenexa, USA). Other complementary tests include detection in the CSF of traces of the pathogenic agent by 16S rRNA PCR and sequencing analysis (SensiFAST™ SYBR Hi-ROX kit; Meridian Bioscience, Inc., Cincinnati, OH, US., and sequences were identified using the Blast algorithm in the National Center for Biotechnology Information [NCBI] database), detection of *S. pneumoniae* antigen in CSF (BinaxNOW S. pneumoniae Antigen Card (BinaxNOW; Abbott, Chicago, USA), *Neisseria meningitidis* antigen (latex agglutination Wellcogen™ *Neisseria meningitidis;* Thermofisher, Waltham, USA), pathogen isolation in blood cultures (BACTEC™ FX; BD®; NYSE, USA), and detection of *S*. *pneumoniae* antigen in urine (BinaxNOW S. pneumoniae Antigen Card (BinaxNOW; Abbott, Chicago, USA). Conventional viral detections were performed by real-time PCR for HSV1/2, CMV, HHV-6, VZV (Nanogen Advanced Diagnostics; Palex®, Barcelona, Spain), and Enterovirus (OneStep RT-PCR Kit; QIAGEN®, Hilden, Germany). FA was performed in all cases, but conventional tests were not always available due to insufficient sample volume. Complementary tests were performed, whenever feasible, based on physicians’ discretion. Results obtained were used to support other clinical, laboratory, and radiological findings to get the final diagnosis.

### Statistical analysis

Categorical variables were described as counts and percentages, whereas continuous variables were expressed as means and standard deviations (SD) or medians and interquartile ranges (IQRs). All analyses were performed with SPSS software (version 25.0; SPSS, Inc., Chicago, IL). The sensitivity, specificity, and predictive values of the technique were based on the final diagnosis of the episode, as determined by the investigators.

### Ethics approval

This observational study was conducted in accordance with the Declaration of Helsinki and was approved by the Ethics Committee Board of the Hospital Clinic of Barcelona (HCB/2022/0943). To protect personal privacy of patients, identifiable information in the database was encrypted for each patient. Informed consent was waived, as no intervention was involved, and no patient-identifiable information was included.

## Results

### Cohort characteristics, clinical presentation, outcomes, and CSF characteristics

During the study period, FA multiplex real-time PCR was performed in 313 patients with suspicion of ME. Table 1 shows the cohort characteristics and clinical presentation of the episodes. Overall, 123 (39.3%) patients had some known immunosuppression and 18 (5.8%) patients had neutropenia. Most patients presented with altered mental status (65.2%) and fever (61%), while only 38 (12.1%) had neck stiffness. ICU admission was required in 135 (43.1%) patients, and 30-day mortality was 11.8%.

**Table 1:** Cohort characteristics and clinical presentation

	All episodes, N = 313
Demographics	
Age, years, median (IQR)	56 (18–96)
Male sex, n (%)	169 (54)
Comorbidities	
Solid tumor, n (%)	25 (8)
Hematological malignancy, n (%)	49 (15.7)
CAR-T recipients, n (%)	13 (4.2)
Hematopoietic stem cell recipients, n (%)	10 (3.2)
Solid organ transplant, n (%)	8 (2.6)
Prior corticosteroids, n (%)	11 (3.5)
HIV, n (%)	26 (8.3)
Autoimmune disease, n (%)	12 (3.8)
Hepatic cirrhosis, n (%)	1 (0.3)
Neutropenia, n (%)	18 (5.8)
Any immunosuppression^a^, n (%)	123 (39.3)
Clinical conditions	
Fever, n (%)	191 (61)
Headache, n (%)	92 (29.4)
Neck stiffness, n (%)	38 (12.1)
Altered mental status, n (%)	204 (65.2)
Seizures, n (%)	55 (17.6)
Focal neurologic deficit, n (%)	77 (24.6)
Antibiotic treatment prior to lumbar puncture, n (%)	119 (38)
Outcomes	
Final meningoencephalitis diagnosis	79 (25.2)
ICU admission, n (%)	135 (43.1)
30-day mortality, n (%)	37 (11.8)

*IQR* interquartile range, *CAR-T* chimeric antigen receptor T, *HIV* human immunodeficiency virus, *ICU* intensive care unit

^a^Including those patients with any of the prior factors

Table 2 detailed the characteristics of the analyzed CSF. Overall, 154 (49.8%) patients presented high proteins in CSF, and 168 (53.7%) had pleocytosis. Conversely, hypoglycorrhachia was present in only 33 (10.6%) patients.

**Table 2:** Cerebrospinal fluid characteristics

CSF characteristics	All Episodes, N = 313
Glucose level, mg/dL; median (IQR) (n = 310)	68.5 (55–85.3)
Hypoglycorrhachia (< 40 mg/dL); n, %	33 (10.6)
Protein level, mg/dL; median (IQR) (n = 309)	600 (403–1013)
High protein levels (> 600 mg/dL); n, %	154 (49.8)
ADA level, U/L; median (IQR) (n = 197)	8 (6–13)
High ADA levels (> 10 U/L); n, %	71 (36)
LDH level, U/L; median (IQR) (n = 135)	28 (22–48)
High LDH levels (> 40 U/L); n, %	44 (32.6)
Lactate level, mg/dL; median (IQR) (n = 66)	22 (15–34)
High lactate levels (> 35 mg/dL); n, %	15 (22.6)
Red blood cells, cells/mm^3^; median (IQR); n = 302	40 (2–320)
Leukocytes, cells/mm^3^; median (IQR) (n = 309)	12 (2–60)
Pleocytosis (≥ 10 leukocytes/mm3); n, %	168 (53.7)
Neutrophil percentage^a^; mean (SD) (n = 212)	36.0 (38.6)
Lymphocyte percentage^a^; mean (SD) (n = 212)	53.7 (38.1)

*IQR* interquartile range, *ADA* adenosine deaminase, *LDH* lactate dehydrogenase, *SD* standard deviation

^a^Among those with > 10 leukocytes

A final diagnosis of ME was made in 79 (25.2%) patients. In such cases, 65.8% patients had high proteins in CSF, 89.9% had pleocytosis, and 21.5% had hypoglycorrhachia.

### FA results and comparison to routine methods

Out of the 313 FA tests performed, 84 (26.8%) were positive. Table 3 shows the positive FA results. HSV-1 was the most diagnosed pathogen (10.9%), followed by VZV (5.1%), and enterovirus (2.6%). Most diagnosed bacteria were *S. pneumoniae* (1.9%), *L. monocytogenes* (1.3%), and *S. agalactiae* (1.3%). There were 5 cases with positive polymicrobial results.

**Table 3:** FilmArray results

	All episodes N = 313 (%)	True positives N = 64 (%)	False positives N = 24 (%)
Positive FilmArray result	84 (26.8)	–	–
Bacterial positive FilmArray result	20 (6.4)	14 (21.9)	6 (25.0)
*Streptococcus pneumoniae*	6 (1.9)	4 (6.3)	2 (8.3)
*Listeria monocytogenes*	4 (1.3)	4 (6.3)	0 (0)
*Streptococcus agalactiae*	4 (1.3)	4 (6.3)	0 (0)
*Haemophilus influenzae*	3 (1)	0 (0)	3 (12.5)
*Neisseria meningitidis*	2 (0.6)	2 (3.1)	0 (0)
*Escherichia coli*	1 (0.3)	0 (0)	1 (4.2)
Viral positive FilmArray result	71 (22.7)	48 (75)	23 (95.8)
Herpes simplex virus 1	34 (10.9)	17 (26.6)	17 (70.8)
Varicella zoster virus	16 (5.1)	15 (23.4)	1 (4.2)
Enterovirus	8 (2.6)	7 (10.9)	1 (4.2)
Herpes simplex virus 2	6 (1.9)	5 (7.8)	1 (4.2)
Cytomegalovirus	4 (1.3)	3 (4.7)	1 (4.2)
Human herpesvirus 6	3 (1)	1 (1.6)	2 (8.3)
Fungal positive FilmArray result	3 (1)	2 (3.1)	1 (4.2)
*Cryptococcus neoformans/gattii*	3 (1)	2 (3.1)	1 (4.2)
More than one positive result	5 (1.6)	4 (6.3)	5 (20.8)

There were 136 (43.5%) cases in which both FA and routine methods were performed and a positive result was obtained. In 35 (25.7%) of such cases, there was a lack of agreement between FA and routine methods. There were 13 cases in which the FA yielded a positive bacterial result, but the cultures were negative (3 *S. pneumoniae*, 2 *S. agalactiae*, 2 *L. monocytogenes*, 2 *N. meningitidis*, 1 *E. coli*, 1 *H. influenzae*, and 2 positives for both *S. pneumoniae* and *H. influenzae*). On the other hand, there were 7 cases with a negative FA result, in which the culture was positive for bacterial isolates not included in the multiplex PCR (3 *Mycobacterium tuberculosis,* 2 *Enterococcus faecium,* 1 *Klebsiella pneumoniae*, and 1 *Staphylococcus epidermidis*). There were also 12 cases in which the FA yielded a positive viral result, but routine PCRs were negative (9 HSV-1, 1 CMV, 1 HHV-6, and 1 positive for both HSV-1 and HSV-2). Finally, there were 5 cases where FA was negative, but routine PCRs were positive for a viral infection (all for HSV-1).

### FA performance for the diagnosis of ME

After clinical assessment, of the 229 negative FA results, 214 (93.4%) were considered true negatives, while 15 (6.6%) were considered FN. Most (10 out of 15) of the considered FN cases presented with ME caused by microorganisms not included in the multiplex PCR panel.

Of the 84 positive FA results, 64 (76.2%) were considered true positives, while 24 (28.6%) were considered FP. There were 4 polymicrobial results, in which both true and FP results were obtained. In this sense, all five cases in which the FA yielded a positive polymicrobial result, some or all the results were considered to be a FP. FP results were mainly attributed to HSV-1 (n = 17), followed by *H. influenzae* (n = 3), *S. pneumoniae* (n = 2), HHV-6 (n = 2), CMV (n = 1), enterovirus (n = 1), HSV-2 (n = 1), VZV (n = 1), *E. coli* (n = 1), and *C. neoformans/gattii* (n = 1). Table 4 details all FP cases and their final diagnosis.

**Table 4:** FilmArray false positive cases, final diagnosis, and methodology used to determine it

Case	FA result	Viral PCR	CSF culture	Other micro-biological result	Final diagnosis	Methodology
1	*S. agalactiae* *H. influenzae* *S. pneumoniae* HSV-1 HSV-2	Negative	Negative	*S. agalactiae* (16S PCR)	*S. agalactiae* meningitis	Clinical and microbiological diagnosis
2	*C. neoformans /gatti* Enterovirus HSV-1	HSV-1 Positive	Negative		HSV-1 encephalitis	Clinical and microbiological diagnosis
3	VZV HSV-1	VZV Positive	Negative		VZV encephalitis	Microbiological diagnosis
4	VZV HSV-1	VZV Positive	Negative		VZV encephalitis	Microbiological diagnosis
5	HSV-1	Negative	Negative		Seizures	Clinical diagnosis
6	HSV-1	NP	Negative		Deep brain stimulation device infection	Clinical diagnosis
7	HSV-1	Negative	Negative		Drug overdose	Clinical diagnosis
8	HSV-1	NP	Negative		Stroke	Clinical diagnosis
9	HSV-1	NP	Negative		Stroke	Clinical diagnosis
10	HSV-1	HSV-1 Positive	Negative	Positive cryptococcal antigen in blood, but negative in CSF	Disseminated cryptococcosis without clear CNS involvement	Clinical and microbiological diagnosis
11	HSV-1	NP	Negative		Wernicke encephalopathy	Clinical diagnosis
12	HSV-1	Negative	Negative		ICANS	Clinical diagnosis
13	HSV-1	NP	Negative		Lewy body dementia	Clinical diagnosis
14	HSV-1	Negative	Negative		Drug overdose	Clinical diagnosis
15	HSV-1	Negative	Negative		Non-Hodgkin lymphoma leptomeningeal disease	Clinical diagnosis
16	HSV-1	Negative	Negative		Drug overdose	Clinical diagnosis
17	HSV-1	Negative	Negative	Positive cryptococcal antigen in CSF	Cryptococcal meningitis	Clinical and microbiological diagnosis
18	HSV-1	HSV-1 Positive	Negative	Adenopathy biopsy culture positive for *M. tuberculosis*	Tuberculous meningitis	Clinical and microbiological diagnosis
19	HHV-6	Negative	Negative	Positive *T. gondii* PCR	Cerebral toxoplasmosis	Clinical and microbiological^c^ diagnosis
20	HHV-6	NP	Negative		ICANS	Clinical diagnosis
21	*H. influenzae* *S. pneumoniae* CMV	Negative	Negative		Acute transverse myelitis	Clinical diagnosis
22	VZV	NP	Negative		COVID-19 ARDS	Clinical diagnosis
23	*E. coli*	NP	Negative		Cyclosporine-associated neurotoxicity	Clinical diagnosis
24	*H. influenzae*	NP	Negative	Positive Paramyxovirus PCR	Paramyxovirus meningitis	Clinical and microbiological diagnosis

*FA* FilmArray, *CSF* cerebrospinal fluid, *NP* not performed, *ICANS* immune effector cell-associated neurotoxicity syndrome, *ARDS* acute respiratory distress syndrome

^#^Clinical diagnosis was based in radiological results, clinical evolution, response to treatment, and potential alternative diagnosis. All cases considered to be false positives improved despite the lack of antiviral treatment

Considering a 25.2% of infection prevalence in this cohort, the sensitivity and specificity of FA were 81.0% (95% CI, 70.6%-89.0%) and 89.9% (85.4%-93.4%), respectively. The positive predictive value (PPV) was 73.0% (64.6%-80.1%) and the negative predictive value (NPV) was 93.4% (89.9%-95.7%). After excluding those pathogens not included in the multiplex PCR panel, NPV increased to 97.41% (94.4%-99.1%).

### Episodes without CSF pleocytosis and/or high CSF protein levels

There were 145, and 155 cases in which FA was performed despite the lack of pleocytosis, or high CSF protein levels, respectively. Of those cases without pleocytosis, or high CSF protein levels, 8 (5.5%), and 26 (16.8%), respectively, were finally considered to have presented a viral meningoencephalitis. No bacterial infection was diagnosed without pleocytosis. Only one *N. meningitidis* meningitis was diagnosed without high CSF protein levels.

Table 5 shows sensitivities, specificities, and predictive values of FA regarding CSF characteristics. Remarkably, PPVs as low as 36.9%, and 70.2%, were found in cases with a lack of pleocytosis, or lack of high CSF protein levels, respectively.

**Table 5:** Estimated prevalence, sensitivity, specificity, and predictive values regarding CSF characteristics

	Meningoencephalitis diagnosis, n (%)	Sensitivity % (95% CI)	Specificity % (95% CI)	Positive predictive value % (95% CI)	Negative predictive value % (95% CI)
Overall (n = 313)	79 (25.2)	81.0 (70.6–89.0)	89.9 (85.4–93.4)	73.0 (64.6–80.1)	93.4 (89.9–95.7)
No pleocytosis (n = 145)	8 (5.5)	87.5 (47.4–99.7)	91.3 (85.3–95.4)	36.9 (24.3–51.6)	99.2 (95.3–99.9)
No high CSF protein levels (n = 155)	26 (16.8)	80.8 (60.7–93.5)	93.1 (87.3–96.8)	70.2 (55.0–82.0)	96.0 (91.6–98.1)
Pleocytosis (n = 168)	71 (42.3)	80.3 (69.1–88.8)	88 (80.0–93.6)	83.1 (74.0–89.4)	85.9 (79.1–90.7)
High CSF protein levels (n = 154)	52 (33.8)	80.8 (67.5–90.4)	85.7 (77.5–91.8)	74.3 (63.9–82.5)	89.7 (83.3–93.9)
Both pleocytosis and high CSF protein levels (n = 110)	50 (45.5)	82.0 (68.6–91.4)	82.5 (70.9–90.9)	79.7 (69.3–87.2)	84.6 (75–90.9)

*CI* confidence interval, *CSF* cerebrospinal fluid

## Discussion

The current study describes a large cohort of patients in whom a ME was suspected and the multiplex real-time PCR FilmArray® (FA) ME Panel (BioFire Diagnostics) was performed to identify the etiology. We aimed to evaluate FA performance and its concordance with routine microbiological methods. The most important findings were: (1) FA was frequently performed in patients without high CSF protein levels or pleocytosis; (2) in this setting, over a quarter of patients presented a positive result, mostly due to viral pathogens; (3) in those cases in which both FA and routine methods were performed, there was a substantial lack of concordance (25.7% of cases); (4) FA was associated to a low rate of FN results, but a considerable rate of FP were found, resulting in excellent specificity and NPV, with moderate sensitivity and PPV; (5) in those cases without pleocytosis, or high CSF protein levels, PPV was substantially lower (Table 5).

In our cohort, FA was performed in patients with clinical suspicion of ME, but in which CSF characteristics were frequently only mildly altered. In this context of potential low pre-test probability of ME, a positive FA result was found in 26.8% of the patients, mostly due to viral pathogens. Prior studies regarding patients with suspected ME reported slightly higher numbers of proteins and leukocytes in the CSF samples, but the rates of positivity were similar, ranging from 8 to 25% [5, 9–12]. Similar to our study, most positive FA results were viral pathogens, mainly HSV-1, enterovirus, and VZV, although this varied widely, especially regarding some studies including children. As expected, the most common bacterial pathogen in our cohort and other studies was *S. pneumoniae.*

In our cases, when both routine methods and FA were performed, concordance rates were relatively low (74.3%). These findings contrast with previous studies where overall agreement between techniques was found in over 80–90% of cases [11, 13–15]. The lack of concordance in our study was mainly due to positive FA results with negative routine tests. Regarding bacterial pathogens, this could be explained by the high rates of antibiotic use before lumbar puncture (38% in the whole cohort), potentially causing a negative culture [16, 17]. In this sense, half of patients with positive FA and a negative CSF culture had received antibiotic prior to CSF analysis. Regarding viral pathogens, there were mainly positive FA results that were finally negative in routine methods, but also negative FA results with positive routine PCRs. The potential explanation for this lack of agreement for viral pathogens is unclear since both methods are real-time PCRs, although they use different primers/probes and are run on different platforms. One potential limitation of FA is that is a closed, qualitative technique, in which only the melting curve is available, but amplification curves or cycle thresholds are not available. This information could be helpful to interpret discordant or uncertain cases.

After the final clinical and microbiological evaluation of the episode, the performance of the FA was overall good. Like previous reports [7, 8], high specificity and NPV (89.9% and 93.4%, respectively) were obtained. Moreover, many of the few false negative results were ME cases caused by pathogens not included in the multiplex PCR panel, which is an expected limitation of the technique. On the other hand, the rates of FPs were high (25.8%) resulting in a relatively low PPV (73%). This is somehow higher to other studies with FA, reporting FP rates around 10–30% compared to reference tests combined with clinical and analytical data [8, 10–12]. False positive results by bacterial targets such as *H. influenzae* and *S. pneumoniae* (which accounted for most bacterial FP results in our cohort) could be explained by potential specimen contaminations at the time of collection or processing by colonized professionals. However, most FP results in our cohort were due to HSV-1, which contrasts with most studies reporting bacterial pathogens as the most frequent cause of FP [7, 8, 10]. In fact, half of the positive FA results for HSV-1 were considered FPs, with previous studies reporting rates of FP HSV-1 ranging from 5 to 50% among positive cases, but with significantly lower cases [7, 10–12]. One potential hypothesis would be that positive HSV-1 from the blood instead of CSF is being detected due to hematic contamination of the sample [8, 11]. Interestingly, out of the 24 FP results, only 3 had no red cells in the sample, and 12 of them had more than 100 red cells/mm^3^. Unfortunately, none of these FP cases had a blood HSV-1 viral load performed at a time close to the lumbar puncture. Another explanation would be that all positive FA results were assessed and prospectively followed by an Infectious Diseases specialist, potentially allowing for antiviral stopping and FP detection whenever the other tests and clinical picture were not suggestive of herpes encephalitis. Finally, the low PPV in our study was mainly driven by the high rates of only mildly or not altered CSF characteristics, which is known to decrease the FA performance [5, 8], particularly when the pre-test probability of ME is low. In this sense, PPV in those patients with either lack of pleocytosis, or normal CSF proteins, was remarkably low. This is challenging since many patients presenting with ME suspicion will actually have these only mildly altered CSF characteristics. This is especially important in severely immunosuppressed patients with profound neutropenia, as they may develop CNS infections without pleocytosis. In such a context, FA could still be useful due to its high NPV [18, 19], but confirmation of positive results (with routine methods, radiology, etc.) is imperative.

The strength of this study is that it thoroughly analyzes all clinical cases to evaluate the performance of FA in a real-life setting. However, this study has some limitations that should be acknowledged. First, this is a single-center study reporting a moderate number of cases over four years. Second, routine analysis of all samples was not available due to insufficient sample volume. Third, complementary tests were not performed systematically but based on the physicians' discretion. Finally, as already exposed, FA performance was limited by the fact that it was frequently performed in patients with normal or slightly altered CSF characteristics.

In conclusion, FA is a useful technique in cases of suspected ME, providing fast results with high NPV. This could be helpful to rule out ME and avoid unnecessary treatments. However, FP results are frequent, especially in patients without high CSF protein levels or pleocytosis.

## Data Availability

Data would be available from the corresponding author under reasonable request.
